# Wastewater genomic surveillance identifies the emergence of the SARS-CoV-2 JN.1 lineage in urban settings of Gujarat

**DOI:** 10.3389/fmicb.2026.1791178

**Published:** 2026-06-11

**Authors:** Jill Gada, Pranav C. Patel, Harshal Purohit, Chaitanya Joshi, Madhvi Joshi, Priyank Chavda, Snehal Bagatharia, Amrutlal Patel, Bhumika Prajapati

**Affiliations:** Gujarat Biotechnology Research Centre, Gandhinagar, Gujarat, India

**Keywords:** digital PCR, Gujarat, JN.1, pandemic monitoring, SARS-CoV-2, WBE

## Abstract

**Introduction:**

Wastewater based epidemiology (WBE) has become a crucial epidemiological tool to supplement clinical surveillance to monitor the spread of SARS-CoV-2 and variant dynamics on the population level. As viral shedding occurs early and independently of healthcare seeking behavior, WBE provides advanced signals of infection trends and variant emergence.

**Materials and methods:**

This longitudinal, multicenter, quantitative and genomic surveillance study of SARS CoV2 was done using wastewater samples from Ahmedabad, Gandhinagar and Vadodara between January 2023 to July 2024. Digital PCR (dPCR) was used to determine the amount of viral load in 2,130 wastewater samples followed by whole genome sequencing (WGS) of high-titer samples.

**Results:**

The concentration of wastewater viral genome has strong statistical correlation with reported cases of clinical COVID-19 (Pearson correlation coefficient r = 0.898; *p* < 0.001), which serves as a robust predictor. Wastewater showed strongest predictive value at 1-week lag in Gandhinagar and Vadodara, while Ahmedabad showed statistical significance for 2 weeks lag. Notably, abundance-based dominance analysis showed that wastewater monitoring also predicted alterations in dominant variants. In Ahmedabad, JN.1 was detected one week earlier than in clinical samples, while KP.x had a lead time of three weeks in Ahmedabad. Temporal mapping at the mutation level also supported the initial occurrence and maintenance of mutations that define lineages in wastewater even before clinical identification, suggesting that there was a gradual formation of new variants on the population level.

**Discussion:**

This study demonstrates that comprehensive quantitative and genomic wastewater monitoring aids to detect presence of SARS-CoV-2 in community early with proactive understanding of the changes in the most common variants within the community. These results highlight the importance of wastewater monitoring as a sensitive, scalable, and proactive tool of population health in terms of early warning and preparedness of pandemics.

## 1 Introduction

COVID-19 pandemic had major ramification on global health, leading to the profound economic and social consequences, widespread infectious illnesses and deaths. During initial stage of the pandemic, limited clinical testing capacity combined with high proportion of asymptomatic individuals (approximately 30% of all carriers), made it difficult to track virus transmission and accurately evaluate burden of COVID-19 in the communities (Bi et al., 2021; Feng et al., 2021). SARS-CoV-2 primarily affects the respiratory system, however it binds with the ACE2 receptor of the gastrointestinal tract and excreted through fecal excreta (Amereh et al., 2022; Wu et al., 2020). This understanding has positioned wastewater-based epidemiology (WBE) at the forefront of community-level health surveillance systems. Virus particles and fragments excreted thorugh infected individuals in stool can enter sewage system, making wastewater an important sampling source to estimate spread of SARS-CoV-2 and utilized by many researchers across the globe (Ahmed et al., 2020; La Rosa et al., 2020; Lodder and de Roda Husman, 2020; Medema et al., 2020; Randazzo et al., 2020; Rimoldi et al., 2020). Presence of infected cells in gastrointestinal tract support transmission of virus through fecal-oral route (Xiao et al., 2020; Xu et al., 2022).

Wastewater treatment plants (WWTPs) and pumping stations have become supplementary tools as SARS CoV-2 RNA are detected from stool samples in sewage (Cluzel et al., 2022). Wastewater enables estimation of true extent of infection in community by capturing viral shedding from infected individuals regardless of symptoms onset, including shedding that occurs before clinical symptoms occur (Bivins et al., 2020; Hart and Halden, 2020; Peccia et al., 2020; Randazzo et al., 2020; Wu et al., 2022). The prolonged incubation period and virus shedding even from asymptomatic individuals contribute to the rapid spread of the virus without medical detection and containment (Li et al., 2020). Wastewater surveillance provides a near real-time picture of viral disease burden within a community by detecting SARS-CoV-2 RNA in feces and nasal secretion from untreated wastewater (Wu et al., 2020). Wastewater surveillance is scalable, cost-effective, and enables anonymous tracking of community-level infection and disease prevalence (Bivins et al., 2020; Keshaviah, 2017; Kumar et al., 2021). Implementing wastewater genomic surveillance poses technical challenges due to low viral loads, fragmented RNA, and elevated levels of PCR inhibitors. To overcome the challenges posed by low viral loads, development and utilization of a highly sensitive detection method is crucial. Compared to the gold standard method RT-qPCR, digital PCR (dPCR) is emerging for precisely detecting the rare targets in environmental samples (Li et al., 2018, 2020). Improved detection limit of dPCR makes it a valuable tool for accurately identifying and quantifying even miniscule amounts of viral genetic material in a sample.

Whole-genome sequencing (WGS) is quite challenging with environmental samples like sewage water due to the mixture of viral lineages, nucleic acid fragmentation, difficulties in sample processing involved in the targeted sequencing and library preparation. However, wastewater-based genomic surveillance can serve as a cost-effective alternative or complementary approach to clinical genomic surveillance, providing valuable information on circulating known or unknown virus lineages within a specific area. Wastewater testing has demonstrated high sensitivity and specificity in detecting SARS-CoV-2 variants, including emerging ones (Crits-Christoph et al., 2021; Godinez et al., 2022; Weidhaas et al., 2021). Its ability to provide parallel and complementary snapshots of community health make WBE a crucial tool to perform environmental surveillance in various countries. Wastewater detection of SARS-CoV-2 has a correlation with COVID-19 incidence, often preceding the increase in clinical cases by 1–3 weeks (Keshaviah, 2017). Additionally, wastewater surveillance has been utilized to detect regionally prevalent variants of SARS-CoV-2 (Crits-Christoph et al., 2021). The establishment of wastewater surveillance networks for routine monitoring of pathogens has proven to be a valuable tool for providing early warning signals of community outbreaks, monitoring pandemic dynamics (Keshaviah, 2017), informing public health interventions (Keshaviah, 2017), and detecting individuals infected with variants of concern in community. Given the ongoing evolution of SARS-CoV-2 and emergence of new variants with increased transmissibility or disease severity, timely and accurate quantification of locally prevalent variants is crucial for effective public health measures (Keshaviah, 2017). In this regard, the present comprehensive study aimed to (a) Carry out surveillance of SARS-CoV-2 in the sewage networks of three major cities of Gujarat using digital PCR (dPCR); (b) longitudinal and monthly correlation analysis of SARS-CoV-2 viral loads in wastewater with reported active COVID-19 cases in Ahmedabad, Gandhinagar, and Vadodara; and (c) next-generation sequencing (NGS)–based genomic surveillance of SARS-CoV-2 in wastewater for identification and temporal tracking of circulating variants, and their comparative correlation with contemporaneous clinical SARS-CoV-2 variant data.

## 2 Materials and method

### 2.1 Sampling locations

**Figure 1:**
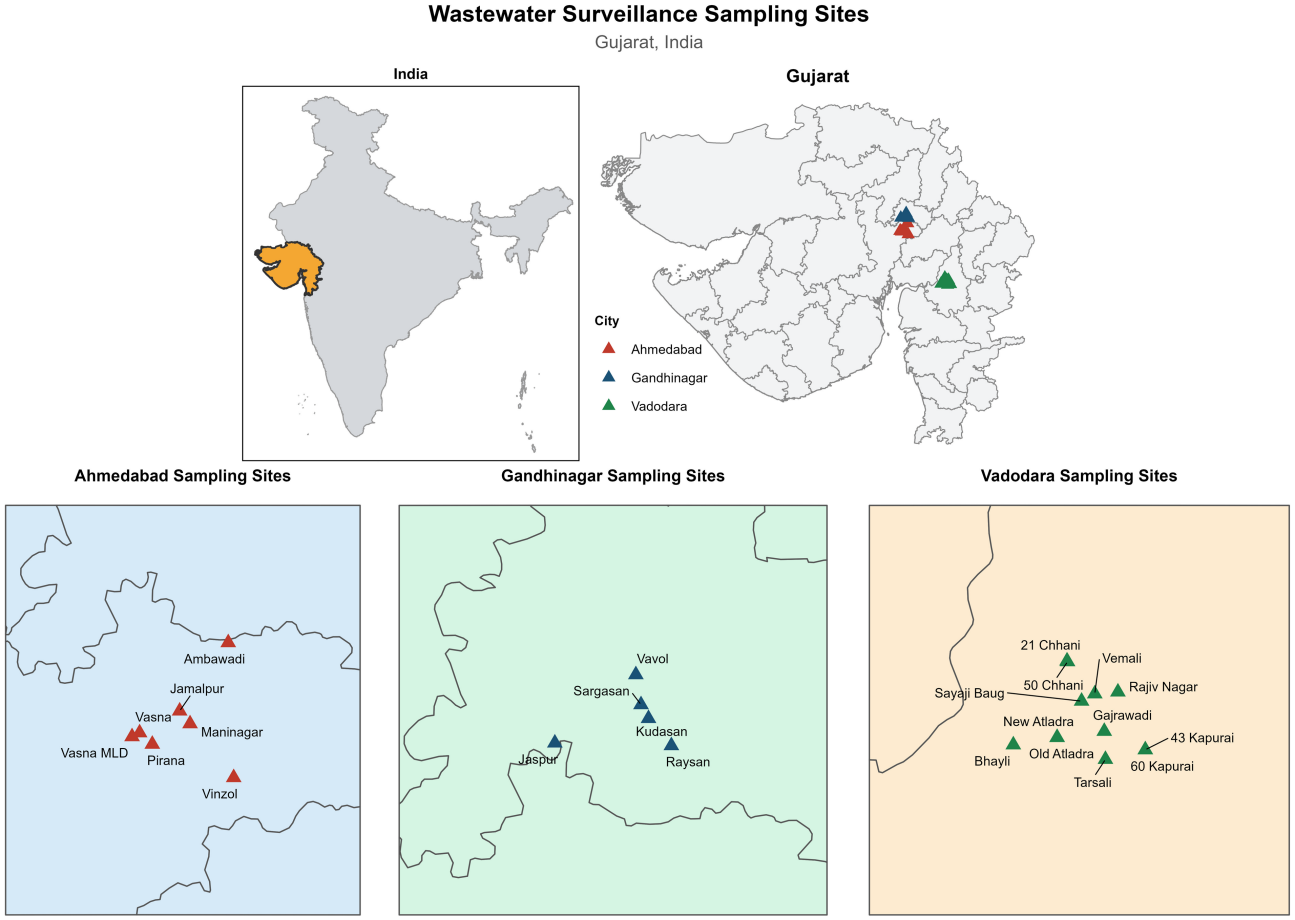
Sampling locations across three cities of Gujarat including seven sites in Ahmedabad city, twelve sites in Vadodara, and five sites in Gandhinagar.

Three major urban cities in the Gujarat state- Ahmedabad, Vadodara, and Gandhinagar were selected for wastewater-based surveillance of SARS-CoV-2. According to the 2011 India Census, Ahmedabad and Vadodara are the first and third most populous cities in the state, with estimated populations of approximately 8.4 million and 5.3 million, respectively. Gandhinagar, the state capital and a major administrative hub housing numerous government offices, with a population of approximately 0.3 million, was also included in the study. Wastewater samples were collected from multiple sewage treatment plants (STPs) and sewage pumping stations (SPSs) across the three cities, comprising seven sites in Ahmedabad, five in Gandhinagar, and twelve in Vadodara. Detailed information on the sampling locations is provided in Supplementary Table S1 and illustrated in Figure 1. Collectively, the selected sites represent catchment areas covering approximately 70–80% of the population in each city.

### 2.2 Wastewater sample collection

Raw, untreated influent wastewater samples were collected from inlets of each site during peak morning hours to capture the maximum amount of virus shed through biological excreta such as feces, urine and saliva. The sewage samples from each selected site were collected using the grab hand sampling method once in a week in 250 mL of sterile polypropylene bottles as described in our previous studies (Kumar et al., 2020). The samples collected were immediately transported from site to the laboratory by maintaining cold storage and standard transportation conditions. The processing and analysis of samples were carried out in the research laboratory of Gujarat Biotechnology Research Centre (GBRC), Gandhinagar, Gujarat.

### 2.3 Virus enrichment and RNA extraction

The samples were further processed for concentration of the virus using previously optimized PEG8000 and NaCl based approaches as described in our previous paper (Kumar et al., 2021). After transferring 30 mL of wastewater sample to 50 mL centrifuge tube, it was subjected to centrifugation at 4000xg for 30 min at 4 °C. The resultant supernatant was filtered using hydrophilic MF-Millipore® mixed cellulose esters (MCE), type GS, pore size 0.22 μm, 25 mm syringe filters (Merck Millipore, Germany) and 25 mL of filtrate was collected in sterile centrifuge tubes. The filtrates were treated with 0.437 g of NaCl (17.5 g/L) and 2 g of PEG-8000 (80 g/L) (Srivastava et al., 2021), and incubated overnight at 4 °C, 100 rpm in a shaking incubator. The enriched samples were then subjected to centrifugation at 14,000xg at 4 °C for 90 min. After discarding the supernatant, the pellets were resuspended in 300 μL of nuclease-free water. The dissolved pellets were collected in 1.5 mL microcentrifuge tubes and stored at −40 °C until further processing or RNA extraction. The viral RNA was extracted using QIAamp Viral RNA mini Kits (QIAGEN, Germany) by adding a MS2 phage control (Thermo scientific, USA) to check the efficiency of the extraction process.

### 2.4 Quantitative estimation of SARS-CoV-2 load using digital PCR

The isolated RNA was subsequently analyzed to quantify the viral load using the QIAcuity One-5 plex digital PCR system (QIAGEN, Germany). The concentration of primers and probes and thermal cycling conditions were optimized for the CDC approved N1 and N2 assays (Tiwari et al., 2022). The assay was carried out using the QIAcuity One-Step Viral RT-PCR Kit, and QIAcuity Nanoplate 8.5 k 24-well (QIAGEN, Germany).

A 12 μL reaction system was used for the assay, consisting of 3 μL of 4x QIAcuity one-step viral RNA master mix, 1.2 μL of 10x SARS-CoV-2 N1 + N2 assay mix (Supplementary Table S2) and 0.12 μL of 100x RT mix. Total 8 μL of extracted RNA was taken as a template. After preparation of a 12 μL reaction mixture in 0.2 mL PCR tube, it was transferred into nanoplate and loaded to the QIAcuity One-5 plex digital PCR system (QIAGEN, Germany). All the samples were run in replicates along with positive, negative and non-template controls. The automated workflow after plate loading includes (i) partitioning (ii) reverse transcription at 50 °C for 30 min, and RT enzyme inactivation at 58 °C for 10 min were the cycling conditions used in the PCR amplification. 40 cycles of 95 °C for 5 s and 60 °C for 30 s follow the initial 2 min of denaturation at 95 °C. (iii) Imaging in the green channel with fluorophore dye as Fluorescein amidite (FAM). Data analysis was done using QIAcuity Suite version 1.1.3 (QIAGEN, Germany), in which quantity was expressed as genome copies per μL (GC/μL) of the reaction mixture. Further they were calculated as GC per liter of wastewater samples (Keshaviah, 2017).

### 2.5 Process recovery analysis and LOD determination

The wastewater samples of were spiked with known amount of MS2 bacteriophage control (10 μL with total 2*103 copies) before processing of the samples. Following that, all samples were processed for virus enrichment, RNA extraction and dPCR assay as mentioned in the methodology section 2.4. The MS2 specific primer and probe in 0.2 μM final concentration was used and sequence were MS2 5′- TGGCACTACCCCTCTCCGTATTCACG-3′ (forward), MS2 R 5’-GTACGGGCGACCCCACGATGAC-3′ (reverse) and probe 5’-FAM-AGCATCATTCCAGGCAC-MGBEQ-3′. The mastermix preparation and dPCR assay cycling conditions were followed as mentioned in section 2.4 with Green detection channel by following the suggested imaging criteria of QIAcuity dPCR software suite. The process recovery efficiency (%) was calculated by following formula: Obtained MS2 copies/Spiked MS2 copies*100. Each sample was processed in replicates with appropriate positive, negative and non-template controls (Kumar et al., 2023). The limit of detection (LOD) of SARS-CoV-2 dPCR assay were assessed by 10-fold serial dilutions of viral RNA copies per reaction as a template starting from 12,000 to 0.12. Positive partition signals were observed for each dilution.

### 2.6 Whole genome sequencing

Based on absolute quantification obtained through dPCR, wastewater samples exhibiting high SARS-CoV-2 genome copy numbers (>5,000 genome copies per liter) were prioritized for whole-genome sequencing (WGS) to ensure sufficient viral RNA input and optimal genome coverage. Samples exhibiting lower genome copies/L were also subjected to sequencing to enable the detection of novel and rare circulating variants in the community. Sequencing libraries were prepared using the Illumina COVID-Seq Test with ARTIC v5.4.2 primers (Research Use Only, RUO) kit following the manufacturer’s given protocol and paired-end sequencing on 2 × 150-bp chemistry was performed on the NovaSeq 6,000 platform (Illumina, USA) using the S4 Reagent Kit (200 cycles).

### 2.7 Bioinformatics analysis

The generated sequencing reads were subjected to downstream bioinformatics analysis using the Freyja pipeline, which deconvolutes mixed SARS-CoV-2 populations in wastewater by leveraging single-nucleotide variant (SNV) frequencies and sequencing depth across the viral genome (Lamba et al., 2023). Wastewater samples with at least 1,000,000 reads and phred score of 30 (Q30) or higher were further downstream analysis. The trimmed reads were mapped to the SARS-CoV-2 Wuhan-Hu-1 reference genome (NC_045512.2) using BWA-MEM. The generated SAM files were converted to BAM, followed by indexing using SAMtools and the variant calling was performed using iVar. Depth files and variant files were generated for further analysis. Lineage deconvolution was done using the Freyja pipeline (v1.4.8) developed by AndersenLab (Karthikeyan et al., 2022). The generated output files provided with relative lineage abundances for each sample.

## 3 Results

### 3.1 Process recovery and sensitivity of dPCR assay

MS2 was spiked as an exogenous control in our study before sample processing. The recovery efficiency was calculated based on the formula provided in the methodology section 2.5. The recovery efficiency for samples was observed between 29.05 to 64.75% which is reported in our previous study and also aligning with other relevant studies (Kumar et al., 2023, Lu et al., 2020). The high abundance of PCR inhibitors in the wastewater samples might affect the recovery efficiency of MS2 phage. The dynamic range of the assay was determined by serial dilutions with genome per copies of 12,000, 1,200, 120, 12, 1.2, and 0.12 per reaction. The qPCR reactions up to 1.2 concentration were quantified with % coefficient of variants (CV) ≤ 20%. The assay demonstrated a good linearity with an R^2^ value of 0.9997 when plot was calculated between the expected copies and measured copies (Supplementary Figure 4).

### 3.2 Temporal trends and case correlation of SARS-CoV-2 in wastewater across Gujarat cities

A total of 2,130, wastewater samples were processed from Ahmedabad, Vadodara and Gandhinagar, and analysed for absolute SARS-CoV-2 quantification using dPCR between January 2023 to July 2024. Across all cities, a pronounced increase in viral genomic load was detected during February–March 2023, with peak concentrations observed in March 2023. Pearson’s correlation analysis revealed a strong and statistically significant association between wastewater viral genome copies and reported active COVID-19 cases (r = 0.898; p < 0.001), demonstrating the utility of WBE as an early indicator for the community infection trends. In Ahmedabad, SARS-CoV-2 RNA copies began to rise in the last week of February 2023 and peaked from mid-March to the second week of April 2023, followed by a steady decline toward the end of the same week, which persisted until September 2023. The highest average viral load was recorded on 16^th^ March (33,910 GC/L) and 23^rd^ March 2023 (31,758 GC/L), preceding an increase in reported active COVID-19 cases by approximately 1–2 weeks. Subsequently, a sharp resurgence in viral genome copies was observed in late October 2023, followed by additional peaks in November and late December 2023. Gandhinagar exhibited comparable temporal patterns, with the highest average viral genome concentrations detected on 16^th^ March (64,040 GC/L) and 6^th^ April 2023 (56,163 GC/L). Following April 2023, viral loads declined progressively until September 2023. A renewed increase was observed in late October 2023, followed by another rise in November and a pronounced peak toward the end of December 2023, which corresponded with an increase in clinically reported cases. In Vadodara, wastewater surveillance revealed the highest average viral loads on 3^rd^ April (98,582 GC/L) and 20^th^ April 2023 (70,013 GC/L). After a decline during mid-2023, viral genome concentrations increased again in October 2023 and remained elevated through November and December 2023. A modest rise was observed in mid-January 2024, followed by fluctuating trends through late January 2024. During the same period, increases in viral genome copies were also observed in Gandhinagar and Vadodara in the last week of January 2024. Month wise average genome copies of SARS-CoV-2 in three different cities is presented in Supplementary Figure 1.

**Figure 2:**
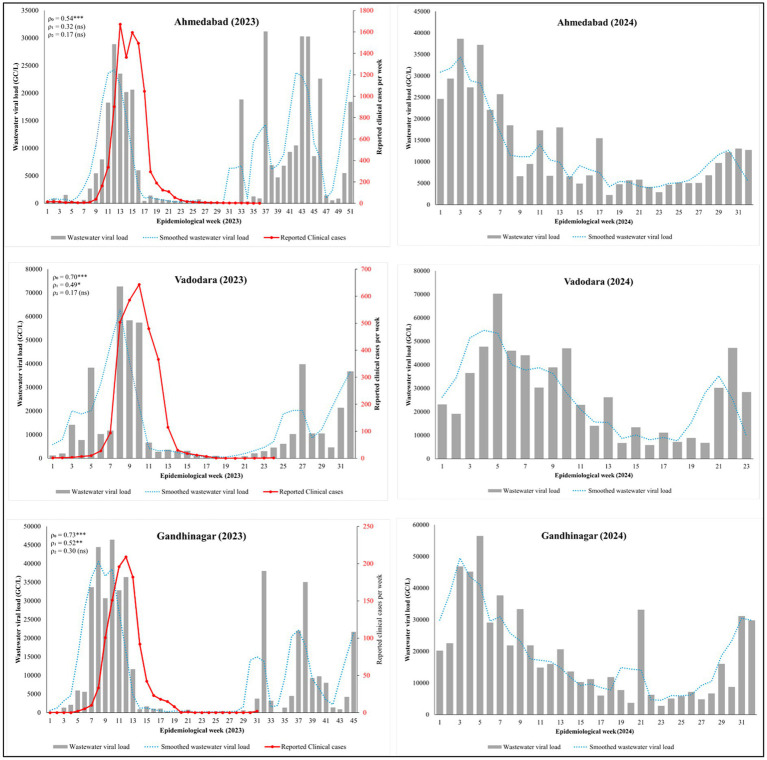
Temporal dynamics of SARS-CoV-2 load in wastewater and COVID-19 confirmed clinical cases in Ahmedabad, Vadodara, and Gandhinagar. The grey bars indicates average viral GC/L of wastewater and orange line indicates weekly average active cases of the city. In Ahmedabad and Gandhinagar, highest viral copies were observed in mid-March, 2023, while in Vadodara the highest viral copies were observed in late March, 2023. In all three cities, there was a peak in active cases also with a lag period of around 2 weeks (10–14 days).

As shown in Figure 2 during February 2024, a slight increase in SARS-CoV-2 RNA concentrations was observed in Ahmedabad, whereas Gandhinagar and Vadodara showed declining trends. Fluctuating viral loads were recorded across all three cities until the end of May 2024, with a notable increase in Gandhinagar during the last week of May. Toward the end of the surveillance period, increased viral genome concentrations were observed in Ahmedabad and Vadodara in late July 2024, while Gandhinagar exhibited a decline during the same interval. Trends in reported active COVID-19 cases closely mirrored wastewater viral load dynamics. Low-level transmission persisted even during periods of reduced wastewater viral concentrations; however, a marked increase in active cases was observed from early March 2023, particularly in Ahmedabad. Peak active case counts were reported in April 2023, followed by a gradual decline by mid-May. A renewed increase in active cases was observed across all three cities in December 2023, coinciding with the emergence of the JN.1 variant. Pearson correlation was used to evaluate the overall linear relationship between wastewater viral load and reported cases using pooled longitudinal data across cities and sampling weeks to assess the overall linear relationship between wastewater viral load and reported clinical cases. Spearman rank correlation was applied for city-specific lag analysis, as it provides a robust non-parametric measure that does not assume normality and is suitable for smaller datasets (Schober et al., 2018).

Table 1 provides a summary of the Spearman correlation coefficients of the weekly wastewater SARS-CoV-2 viral load with reported clinical COVID-19 cases in three cities at various time offsets in 2023. Close temporal agreement between wastewater viral concentrations and reported cases was observed to be statistically significant in Gandhinagar (r = 0.733, *p* < 0.001) and Vadodara (r = 0.702, *p* < 0.001) and moderate but significant in Ahmedabad (r = 0.539, *p* < 0.001). One-week lag wastewater viral load was still found to be strongly allied with case counts in clinical cases in Gandhinagar (r = 0.522, *p* = 0.003) and Vadodara (r = 0.485, *p* = 0.016), indicating that wastewater surveillance could give about a one-week warning of amplified community transmission in the cities. Conversely, the lagged one-week correlation in Ahmedabad was weak and not statistically significant (r = 0.324, *p* = 0.054). At a two-week lag, the correlation between wastewater viral load and reported clinical cases was weak and reached statistical significance only in Ahmedabad (r = 0.166, *p* = 0.033).

**Table 1:** Spearman correlation coefficient between weekly wastewater SARS-CoV-2 viral load and reported clinical COVID-19 cases at same-week, one-week and two-week lag during 2023.

City	Lag	Spearman correlation coefficient	*p* value	*N*
Ahmedabad	Same week (t)	0.539	0.001	36
	1-week lag (t + 1)	0.324	0.054	36
	2-week lag (t + 2)	0.166	0.033	36
Vadodara	Same week (t)	0.702	0.001	24
	1-week lag(t + 1)	0.485	0.016	24
	2-week lag (t + 2)	0.170	0.426	24
Gandhinagar	Same week (t)	0.733	0.001	31
	1-week lag (t + 1)	0.522	0.003	31
	2-week lag (t + 2)	0.302	0.099	31

Statistically significant correlation indicates temporal alignment and early warning of potential wastewater surveillance.

### 3.3 Site-wise comparison of mean SARS-CoV-2 viral genome loads within cities

**Figure 3:**
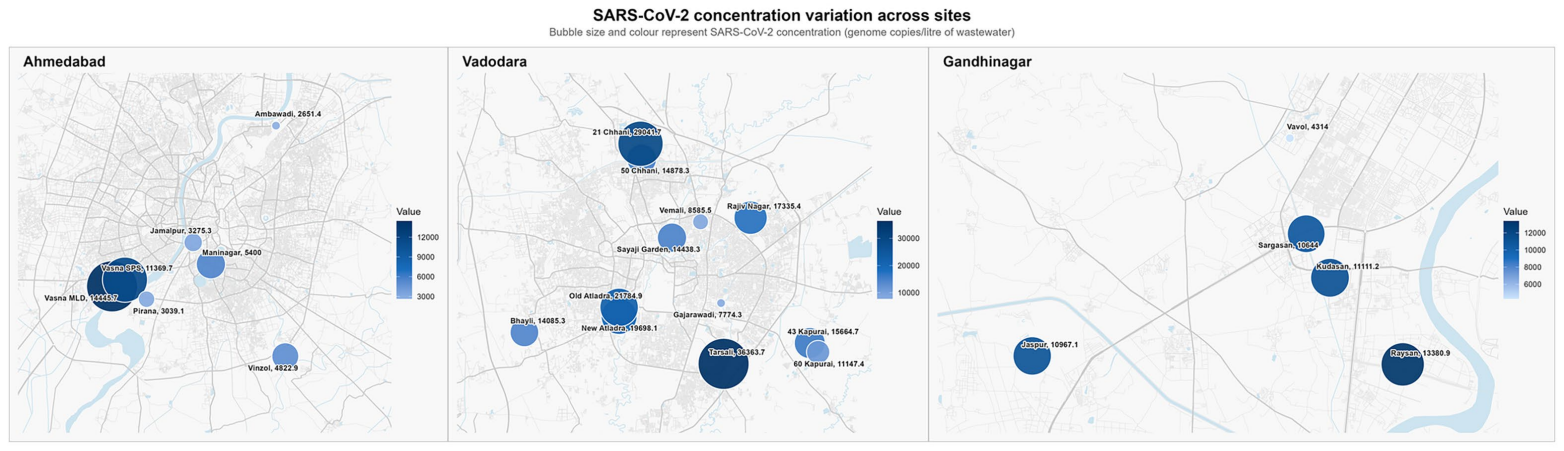
Site-wise comparison of mean SARS-CoV-2 viral genome copies across sampling locations in Ahmedabad, Vadodara, and Gandhinagar over the entire study period (January 2023–July 2024). The sampling sites of three cities have been categorized into hotspots based on the virus copies obtained from that particular site. Sites with dark to light color indicate higher to lower viral RNA copies. In Ahmedabad, Vasna MLD and Vasna SPS showed the highest viral genome copies while in Vadodara, Tarsali and 21 Chhani were the most affected sites. In Gandhinagar city, Raysan has reported highest viral RNA copies across all sampling sites.

The average SARS-CoV-2 genome copies across all sampling sites in Ahmedabad, Vadodara, and Gandhinagar were evaluated throughout the study period. Based on the mean viral genome concentrations, sampling locations were classified as high-signal and low-signal zones (Figure 3). In Ahmedabad, among all sampling sites, Vasna MLD and Vasna SPS exhibited the highest average genome copies per liter and were therefore identified as a high-signal region, followed by Maninagar, Vinzol, and Jamalpur. Ambawadi recorded the lowest average genome copies per liter among all sites in the Ahmedabad region. Similarly, in Gandhinagar, out of the five sampling sites, Raysan showed the highest average genome copies per liter and was identified as the primary high-signal site, followed by Kudasan, Sargasan, and Jaspur. Vavol exhibited the lowest average genome copies per liter among all sites in the Gandhinagar region. In Vadodara, among the twelve sampling sites, Tarsali and 21 Chhani demonstrated the highest average genome copies per liter and were identified as high-signal regions, followed by New Ataladra, Old Ataladra, Bhayli, 60 Kapurai, 43 Kapurai, 50 Chhani, Rajiv Nagar, and Sayajibaugh. Gajarawadi and Vemali recorded the lowest average genome copies per liter among all sites in the Vadodara region.

### 3.4 SARS-CoV-2 variant analysis

**Figure 4:**
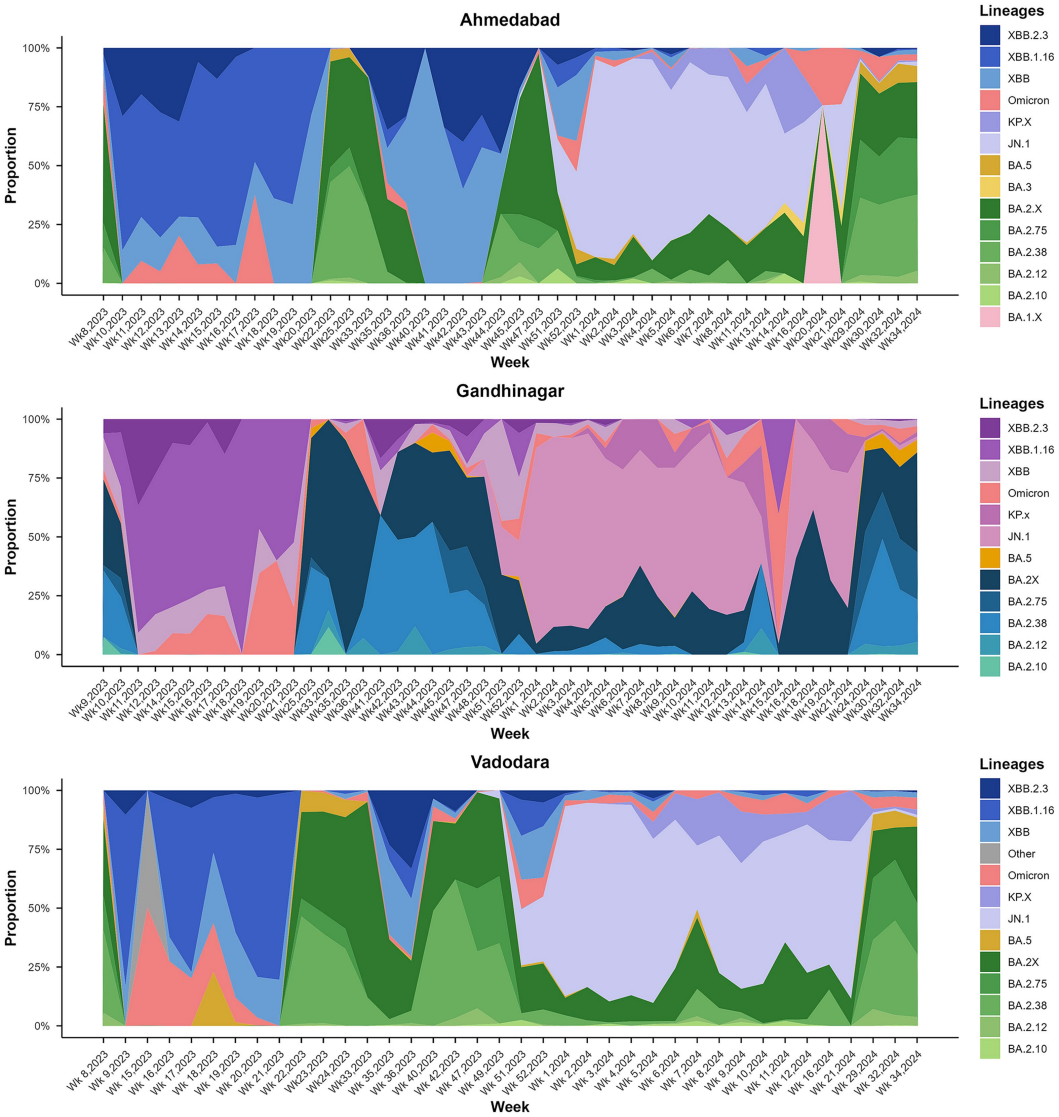
Temporal trends in the lineage composition of SARS-CoV-2 in wastewater of Ahmedabad, Gandhinagar, and Vadodara. Stacked area plots display the relative abundance of the major SARS-CoV-2 lineages found in wastewater samples from January 2023 to July 2024 through whole-genome sequencing. The colored portions denote the percentage of a particular origin within a particular epidemiological week with the numbers being normalized to 100 percent. The plots show patterns of replacement of lineage and co-circulation between the three cities, XBB-derived lineages into BA.2-related ones, and the following appearance and dominance of JN.1 and KP.x lineages. The time changes in the changes in DNA lineages emphasize that there is a spatial heterogeneity in variant circulation, and that wastewater surveillance of genome variants can detect early-stage changes and changes at the population level of predominant SARS-CoV-2 forms.

The sequencing reads generated from all samples were aligned to the SARS-CoV-2 reference genome (NC_045512.2). A total of 272 wastewater samples with viral loads exceeding 5,000 genome copies per liter (Ahmedabad: 92; Gandhinagar: 83; Vadodara: 97) were subjected to sequencing on the Illumina NovaSeq6000 platform. Of these, 246 samples achieved greater than 50% genome coverage and were prioritized for downstream genomic analysis. However, later we analyzed remaining samples with less than 5,000 GC/L also to trace the novel or rare variants circulating in the community. The viral concentration to genome coverage plot for each analyzed sample is shown in Supplementary Figure 5. The Freyja pipeline was employed to estimate the relative abundance and diversity of SARS-CoV-2 lineages in wastewater samples. Analysis identified over 400 distinct lineages, of which approximately 47 were classified as dominant based on relative abundance. Compared to conventional variant-calling approaches, Freyja enabled the detection of additional major and minor variant mixtures that were not resolved by traditional pipelines, highlighting its enhanced sensitivity in complex wastewater matrices. Notably, evidence of co-circulating lineage mixturewas observed in sewage samples, with mutations corresponding to variants also reported in clinical datasets. During the study period, the most prevalent lineages in wastewater were XBB.1.16 sub-lineages, specifically XBB.1.16.4, XBB.1.16.5, and XBB.1.16.1, with approximate mean abundances of 18, 17, and 15%, respectively. In February, BA.2.38 emerged as the dominant lineage (~19%), followed by a gradual decline in March. Abundances of 29% & 24% were prevalent during the months of March and April, respectively followed by XBB.1.16 with 13% in March and 17% in April & XBB.1.16.4 with 11% in March, 12% in April. However, XBB.1.16.4 was the dominant lineage with 48% genomic prevalence in May followed by XBB.1.16.5 & XBB.1.22.2 with 21% genomic prevalence (Figure 4). Since June 2023 BA.2.38 variant of SARS-CoV-2 was seen to be most prevalent with around 22% abundance. In the month of November, a new novel variant of SARS-CoV-2 identified as JN variant was found to be circulating in wastewater samples in minor proportions. In the month of December, the sequencing of wastewater samples revealed the presence of JN sub-lineages such as JN.1, JN.1.1 and JN.1.3 in major proportions in all the three cities.

Whole-genome sequencing (WGS) analyses of wastewater and clinical samples from these major Gujarat cities revealed dynamic changes in SARS-CoV-2 variant circulation over time. Between March and May 2023, the parent lineage XBB.1.16 and its sub-lineages predominated across all three cities. From June to October 2023, BA.2.38 and BA.2. X variants were detected in higher abundance, replacing earlier XBB lineages. During November–December 2023, JN.1variants began to appear in minor frequencies in Ahmedabad and increased in abundance through March 2024, while JN.1, BA.2.74, and XBB lineages were concurrently predominant in Gandhinagar. In Vadodara, BA.2. X and JN.1, were the major circulating lineages during this period. The FLiRT variant (KP.2) was first detected in Vadodara wastewater samples in February 2024 and co-circulated with BA.2. X and JN.1 until March 2024. From April to July 2024, co-circulation of JN.1, BA.2.38, and BA.2. X lineages were consistently observed across the three cities, with BL.1 and BL.1.3 lineages showing higher abundance in Ahmedabad during July 2024. These patterns highlight temporal and spatial diversity in SARS-CoV-2 lineage dynamics across urban regions of Gujarat.

Approximately 1765 numbers of clinical SARS-CoV-2 genomic sequences from three studied cities (Ahmedabad, Gandhinagar, Vadodara) of Gujarat submitted on GISAID by GBRC were used for analysis from the period of February 2023 to August 2024. However, GISAID data for Gandhinagar was not consistent. A total of 60 different lineages were revealed from the GISAID dataset, with XBB.1.16 being the most dominant, accounting for 55.7% of genomes, followed by XBB.1.16.1 with 8% and XBB.2.3 with 6% of genomes. Apart from these other lineages that were seen dominating in the clinical samples were BA.2.38, BA.2.74 during June 2023 to October 2023. In December 2023 JN.1 variant was the most prevailing variant found in the clinical cases.

**Figure 5:**
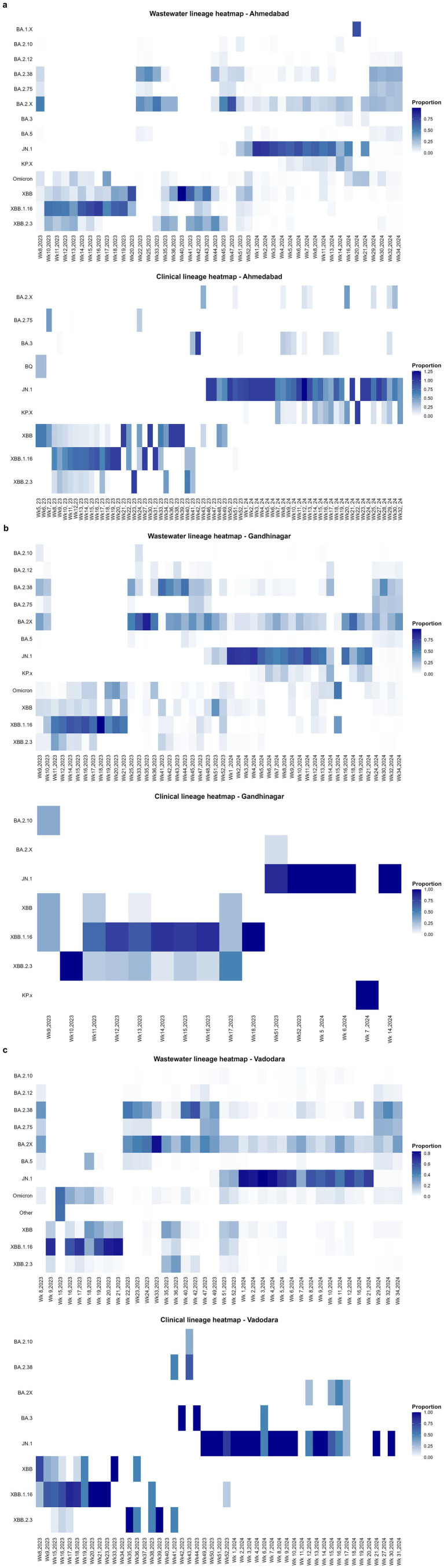
**(a)** Heatmap representing abundance of SARS-CoV-2 lineages detected from wastewater and clinical samples in Ahmedabad. **(b)** Heatmap representing abundance of SARS-CoV-2 lineages detected from wastewater and clinical samples in Gandhinagar. **(c)** Heatmap representing abundance of SARS-CoV-2 lineages detected from wastewater and clinical samples in Vadodara.

SARS-CoV-2 lineages were detected earlier through wastewater surveillance than clinical surveillance in cities both, at level of first detection and in the dominance. JN.1 was initially detected in wastewater in epidemiological week 48 in Gandhinagar three weeks before it was initially identified in clinical samples in week 51. In the same way, KP.x had been found in wastewater previously in Gandhinagar with lead times of three weeks. Although the magnitude of lead time varied by location and lineage, the overall pattern indicates earlier signal capture in wastewater. Figures 5a–c represent variants detected in Ahmedabad, Gandhinagar and Vadodara, respectively.

**Figure 6:**
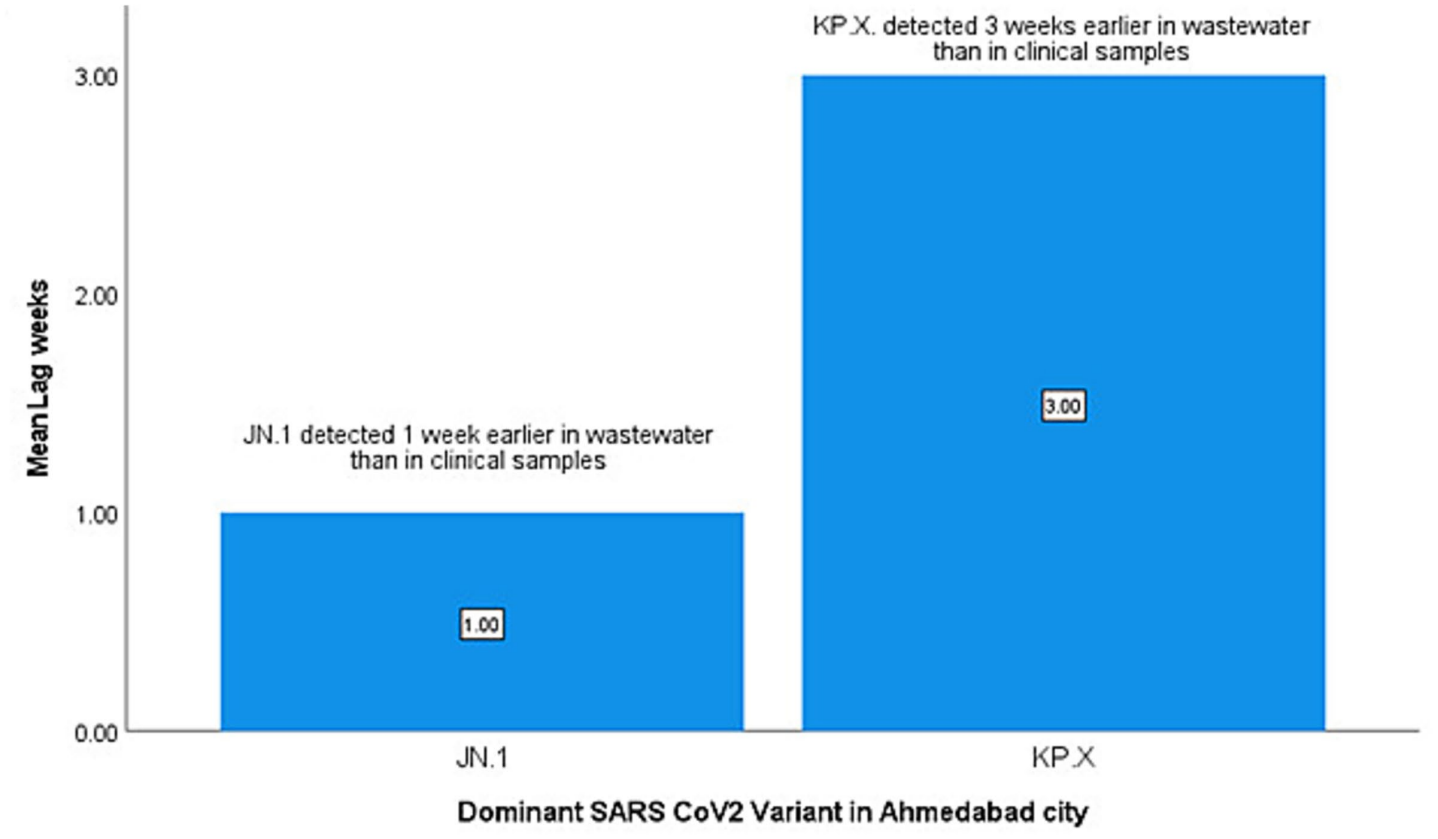
Mean lead time between wastewater and clinical surveillance for dominant SARS-CoV-2 variants in Ahmedabad. Variant dominance was determined from relative abundance data, with lead time calculated as the difference in weeks between the point at which a variant became dominant in wastewater samples and the point at which it became dominant in clinical samples. Positive lead time indicates earlier dominance in wastewater surveillance.

Notably, abundance-based domination analysis also confirms this observation. In Ahmedabad, JN.1 dominated wastewater a week before it dominated clinical samples, whereas the lead time for KP.x was three weeks, as indicated in Figure 6. Collectively, these results indicate that wastewater monitoring is not only capable of identifying new lineages at an earlier stage compared to a clinical sample, but it can also give an early warning of a change in the most common variants in the population.

### 3.5 Temporal mapping of lineage-defining SARS-CoV-2 mutations in wastewater

**Figure 7:**
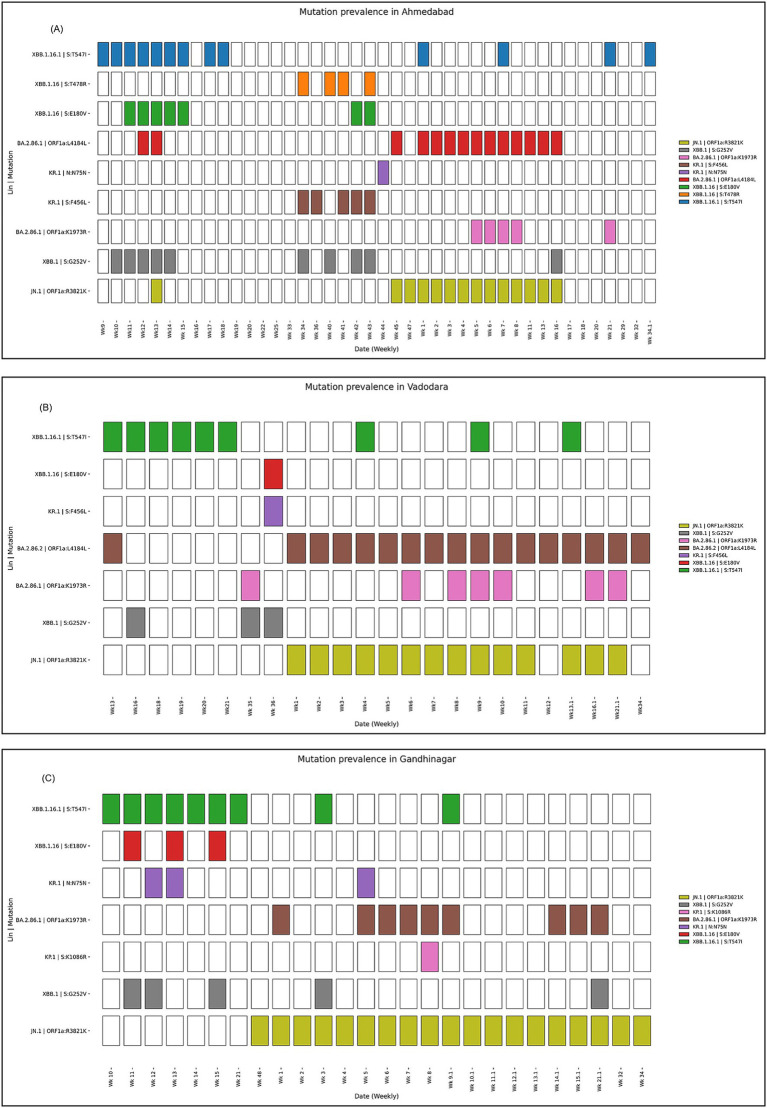
Temporal occurrence of lineage-defining SARS-CoV-2 mutations detected in wastewater samples from **(A)** Ahmedabad, **(B)** Vadodara, and **(C)** Gandhinagar. Each horizontal track represents a specific mutation associated with a known SARS-CoV-2 lineage.

The mutation level temporal maps of wastewater samples from Ahmedabad, Vadodara and Gandhinagar illustrate week wise occurrence of key SARS-CoV-2 lineage defining mutations and provide insights about initial molecular features of new variants. Horizontal tracks (Figure 7) symbolize mutations with particular lineage, and colored blocks show the week when mutation was identified in wastewater. In all three cities, wastewater surveillance showed that the existence of mutations of lineage was already present before detection of clinical lineage. Several mutations associated with XBB- derived lineages were observed in Ahmedabad intermittently and in some cases in consecutive weeks, which indicates community level circulation. In Gandhinagar evidence of mutations related to JN-related lineages was early identified in wastewater with mutation evidence noted several weeks earlier than the associated lineage was found in clinical data. The stability of these mutations over time within consecutive weeks is a manifestation of gradual incorporation of the variant into the population before it is clinically recognized. Other mutations were short lived, suggesting that they were not spread widely or persisted, or that there was minimal circulation. The wastewater samples in Vadodara showed initial molecular marks which could be linked to the lineages of XBB.1.16. The genes of these lineages were detected in wastewater weeks prior to their first discovery in clinical samples. The patterns of mutations that were observed suggest that there is dynamism in available variants in circulation and show how wastewater surveillance can identify new viral diversity sooner as compared to case-based sequencing.

### 3.6 Inter-relational analysis of SARS-CoV-2 lineages in wastewater and clinical samples

The sequencing data obtained was further used to create a Venn diagram (Supplementary Figure 3) which depicts the relative prevalence of the different lineages in wastewater as well as clinical samples. It was observed that there were a total of 11 lineages that were common to clinical samples as well as the wastewater samples of all three cities. The details of the same are given in Supplementary Table S3. There were 23 such lineages that were found in wastewater samples of all three cities but were not found to be present in the clinical samples. It was also seen that there were few lineages that were unique in clinical samples (Supplementary Table S4). Around 4 lineages were only found in Ahmedabad wastewater samples while 7 such unique lineages were seen in Gandhinagar samples and 6 such unique lineages were found in Vadodara. The number of such distinctive lineages found in the clinical cases was 19 lineages.

## 4 Discussion

Wastewater-based epidemiology has become a population level surveillance platform to track the transmission of SARS-CoV-2, especially in locations where clinical tests have varied levels of intensity and the prevalence of asymptomatic cases is under-reported (Keshaviah, 2017). This longitudinal, multi-centric study of quantitative and genomic wastewater surveillance conducted between January 2023 to July 2024 indicates the relevance of wastewater surveillance as an early warning of SARS-CoV-2 circulation and the emergence of a variant. The fact that this has been found to be consistent in three large urban centres in Gujarat supports the fact that wastewater surveillance is a crucial complement to traditional clinical surveillance systems.

The Pearson correlation coefficient revealed a statistically significant association between wastewater genome copies and reported COVID-19 cases (r = 0.8980, *p* < 0.001) (Supplementary Figure 2). This supports the fact that viral load of wastewater effectively represents underlying trends in community transmission (Keshaviah, 2017). Notably, the time composition demonstrated that growths in wastewater viral RNA were anticipated or accompanied by the growths in clinically reported cases, which shows the epidemiological importance of wastewater indications. Wastewater sequencing has demonstrated a high level of success in identifying emerging variants early in the course of infection in a variety of high-impact studies, such as the identification of SARS-CoV-2 transmission and the emergence of variants in the United States and Europe (Diamanti et al., 2024; Jones et al., 2025). Early detection of JN.1, KP.x and XBB related regions in this study extends these observations to Indian urban context. This role is also contributed by mutation level temporal mapping which shows that mutations defining the lineages continue to exist in the wastewater before clinical lineage designation which reflects gradual population level establishment of new variants. A correlation between the genome coverage and the genome copies/L obtained was studied. Pearson’s correlation coefficient was calculated, and it was found to be 0.532 indicating a positive correlation between the genome coverage and the Genome copies/L.

Early research in the United States was the first to establish that wastewater SARS-CoV-2 RNA concentrations can be detected 2–12 days prior to pathologically positive SARS CoV-2 test (Peccia et al., 2020; Schenk et al., 2024). Later research in Asia showed lead times of 14 days (Karthikeyan et al., 2022). The lead time of one to two weeks found in the current study is in line with these findings and promotes the biological nature of early feces shedding of viruses in pre-symptomatic and asymptomatic infection. Lagged correlation analysis also shows the predictive ability of wastewater surveillance at a small forecasting window. Although the same-week and one-week lag correlations were the strongest in the cities, the correlations decreased with increasing lag periods, showing that wastewater signals are the best predictors in the nearest future outbreak. This trend aligns with literature SARS-CoV-2 shedding kinetics (Oordt-Speets et al., 2024) and highlights the utility of wastewater data in short-term preparedness of the public health instead of long-term prediction.

Interestingly, Ahmedabad, Gandhinagar and Vadodara had different strengths of correlations as well as the ideal lead times. The differences are probably due to the city-specific features of population density, sewer network structure, healthcare-seeking behavior, mobility trends, and differences in clinical testing practices. Prior wastewater surveillance studies have also found similar spatial heterogeneity and stated that wastewater indicators need to be understood in context of local epidemiological and infrastructural conditions but not using homogeneous thresholds (Yang et al., 2025). The findings highlight the power of specific city calibration in operationalizing wastewater surveillance in making decisions about human health.

One of the greatest methodological advantages of the current study lies in the fact that the quantification of the viruses was done using digital PCR throughout. RT dPCR has a greater sensitivity and precision and shows resistance to PCR inhibitors than RT-qPCR especially in complex environmental samples like wastewater (Muhammad et al., 2025; Pecoraro et al., 2019). Comparison of RT dPCR with RT-PCR revealed RT dPCR had high sensitivity to detect virus even at low concentration, a factor that is important to maintain surveillance during inter-wave periods (Ahmed et al., 2022). In addition to quantitative surveillance, genomic analysis of wastewater samples was also helpful to reveal the evolutionary characteristics of SARS-CoV-2 at the community level. Wastewater sequencing obtains a composite signal of many co-circulating lineages, an aspect that can be easily overlooked by clinical sequencing methods, which produce single consensus genomes. Using a mixture-conscious deconvolution of the lineage framework, this work was able to determine that a large amount of lineage diversification had occurred and that temporal dynamics of replacements of variants very much reflected the global evolutionary dynamics. The same results have been concluded in other studies, which show that wastewater genomics can effectively capture accurate regional variants circulation and reveal viral diversity which is not well represented in clinical datasets.

This study proves that identification of new variants in wastewater is possible several weeks prior to clinical surveillance. Detection of JN.1, KP.x, and XBB-related lineages prior to clinical reporting supports wastewater genomic surveillance as a sensitive predictive monitoring tool in an urban setting in India. The ability to detect lineage defining mutation at an early stage is substantiated by mutation temporal mapping. In addition to early detection, wastewater monitoring also offered information on changes in the dominant SARS-CoV-2 variants as compared to clinical monitoring. Figure 6 demonstrated that the abundance-based dominance methodology showed that JN.1- dominant variant in Ahmedabad was detected one week prior to its detection clinical samples, and KP.x had a longer lead time of three weeks. This observation from Ahmedabad confirms that wastewater monitoring is no longer restricted to early detection of new lineages but also can be predictive of dynamics of replacement of variants within the population. The previous shift in hegemony of wastewater is probably aggregated viral shedding of both symptomatic and asymptomatic infections, which is able to record patterns of community-wide transmission before it has been translated to clinical testing data. This kind of lead time due to dominance is especially applicable to public health preparedness since it offers prior warning about changes in the circulating variants which can affect transmissibility, immune evasion or intervention measures.

The fact that these mutations are observed to persist in multiple weeks indicates that they are gradually becoming established at the population level only to be picked up by standard clinical surveillance. Lineages found solely in wastewater are probably indicative of infections in asymptomatic or mildly symptomatic people who do not get tested, but lineages found in clinical data sets may indicate low-prevalence clusters or low shedding of feces infections. We compared the efficacy of WBE based variant analysis to clinical genomic epidemiology at the city level by using metadata of GISAID. The sewage data highlights the circulation of XBB.1.16 and its sub lineages, i.e., XBB.1.16.1 and XBB.1.16.5 in similar time frames of the clinical settings. However, sewage samples have shown the early presence of XBB.1.16.5 sub-lineage in the month of March 2023 however the abundance was less due to heterogeneous nature of sewage samples. The clinical samples of all three cities have shown the circulation of XBB.1.16.5 in the month of April and May, 2023, BA.2.38 variant from the month of June till October, In the month of November the wastewater samples showed the prevalence of JN variants in minor proportions about a month prior the detection of JN variants in the clinical samples, which highlights the importance of WBE based genomic surveillance for detection and tracing of the emerging SARS-CoV-2 pathogenic variants in the population. Wastewater and clinical genomic surveillance can give a more detailed view of diverse viruses, a combination of population-level representativeness and individual-level clinical resolution. Our results of variant analysis aligns with the previous report, which observed the circulation of XBB.1.16 and its sublineages during the same study period from Mumbai city (Kadam et al., 2024).

Wastewater surveillance study of viral load of genome also demonstrates its application determining population-wide infectious disease surveillance, with proven track record for polio, hepatitis A, dengue and chikungunya (Monteiro et al., 2024; Whitehouse et al., 2024; Zulli et al., 2024), and holds considerable promise for population-wide surveillance of the COVID-19 pandemic. The identification of high-signal sites across all three cities support the utility of sub-water shed level sampling, although these classifications are solely based on the measured wastewater viral concentration which represents relative viral signal intensity rather than infection burden. Environmental dilution may affect the concentration of viral RNA (Robins et al., 2022), variability in the flow, temperature, and inter-individual variations in shedding (Bertels et al., 2022). In line with these studies, we acknowledge unavailability of data related to wastewater dilution, flow variability and rainfall adjustment as limitations. Also, the positive correlation between the genome coverage and viral load is observed which emphasizes the fact that sufficient levels of RNA depend on the quality of the sequencing. More optimization of the normalization procedures and standard sampling systems will be necessary to improve the readability and comparability of wastewater genomic data. This paper presents substantial data that integrated quantitative and genomic wastewater surveillance is a viable methodology that can be scaled and is sensitive to detecting SARS-CoV-2, identifying new variants before clinical surveillance and defining spatial heterogeneity in infection burden.

## Data Availability

The datasets presented in this study can be found here: https://www.ebi.ac.uk/ena/browser/view/ERP194169.
