# Supplementary material for: Wastewater genomic surveillance identifies the emergence of the SARS-CoV-2 JN.1 lineage in urban settings of Gujarat
Source: Front Microbiol. 2026 Jun 11;17:1791178. doi: 10.3389/fmicb.2026.1791178 (PMC13294091; doi:10.3389/fmicb.2026.1791178)
Supplement: Supplementary file 1 [file Table_1.docx]

Supplementary Material

# Supplementary Figures and Tables

## Supplementary Figures


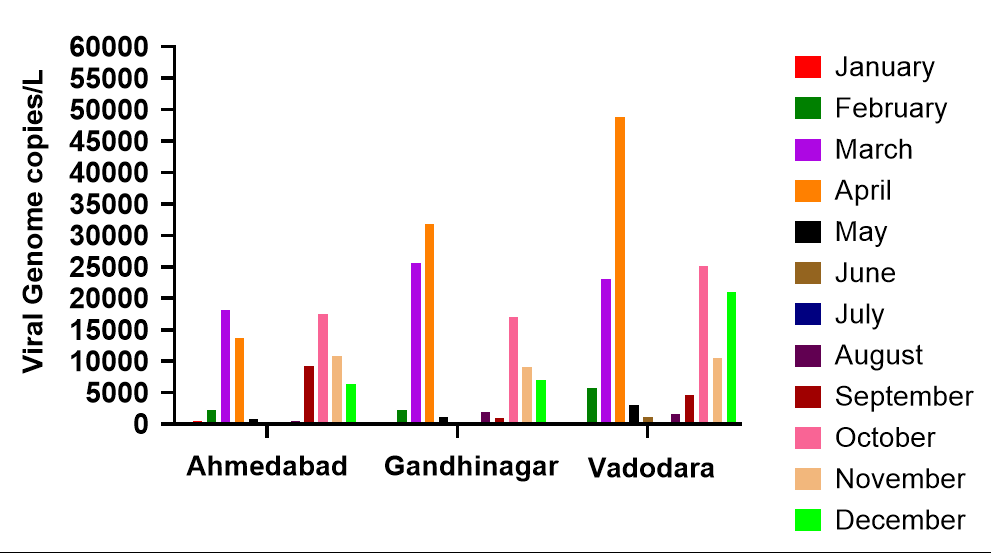


**Supplementary Figure 1.** The above figure represents the average genome copies/L obtained in various months during the study in all the three cities. In the months of January and February the genome copies in all cities were in traces while it was drastically increased during the month of March with highest viral load in the month of April 2023 which corresponds to the peak of the outbreak. During the months of May to August the genome copies were found to be in very minute amounts in all three cities. Since September slight increase in the genome copies was observed followed by a drastic increase in the month of October till December. While comparing viral load amongst three cities, highest viral load was observed in Vadodara during the peak followed by Gandhinagar and Ahmedabad.


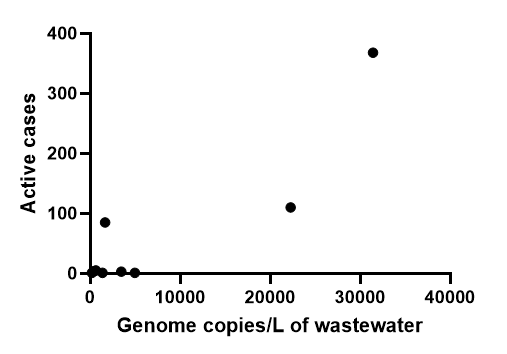


**Supplementary Figure 2**: The figure represents a correlation between the Average Genome copies per liter of sewage to Clinical cases obtained in a particular city. The calculation of correlation coefficient was done using GraphPad Prism V. The positive correlation was observed with r= 0.8980 and statistical test of Pearson correlation was significant with p= 0.0010. This indicated a very strong correlation of the Average copies obtained from wastewater with that of the active cases obtained.


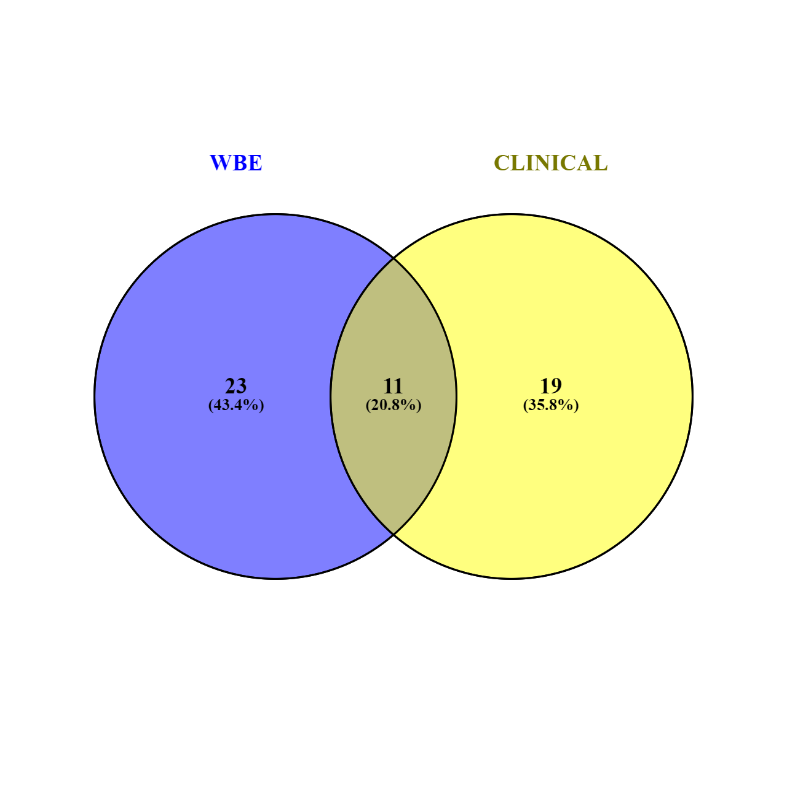


**Supplementary Figure 3:** Venn diagram represents a comparative analysis of the various lineages found in wastewater samples and clinical samples of three cities of Gujarat.

Where, WBE= Wastewater based epidemiology of all three cities

Clinical= Clinical lineages of all three cities


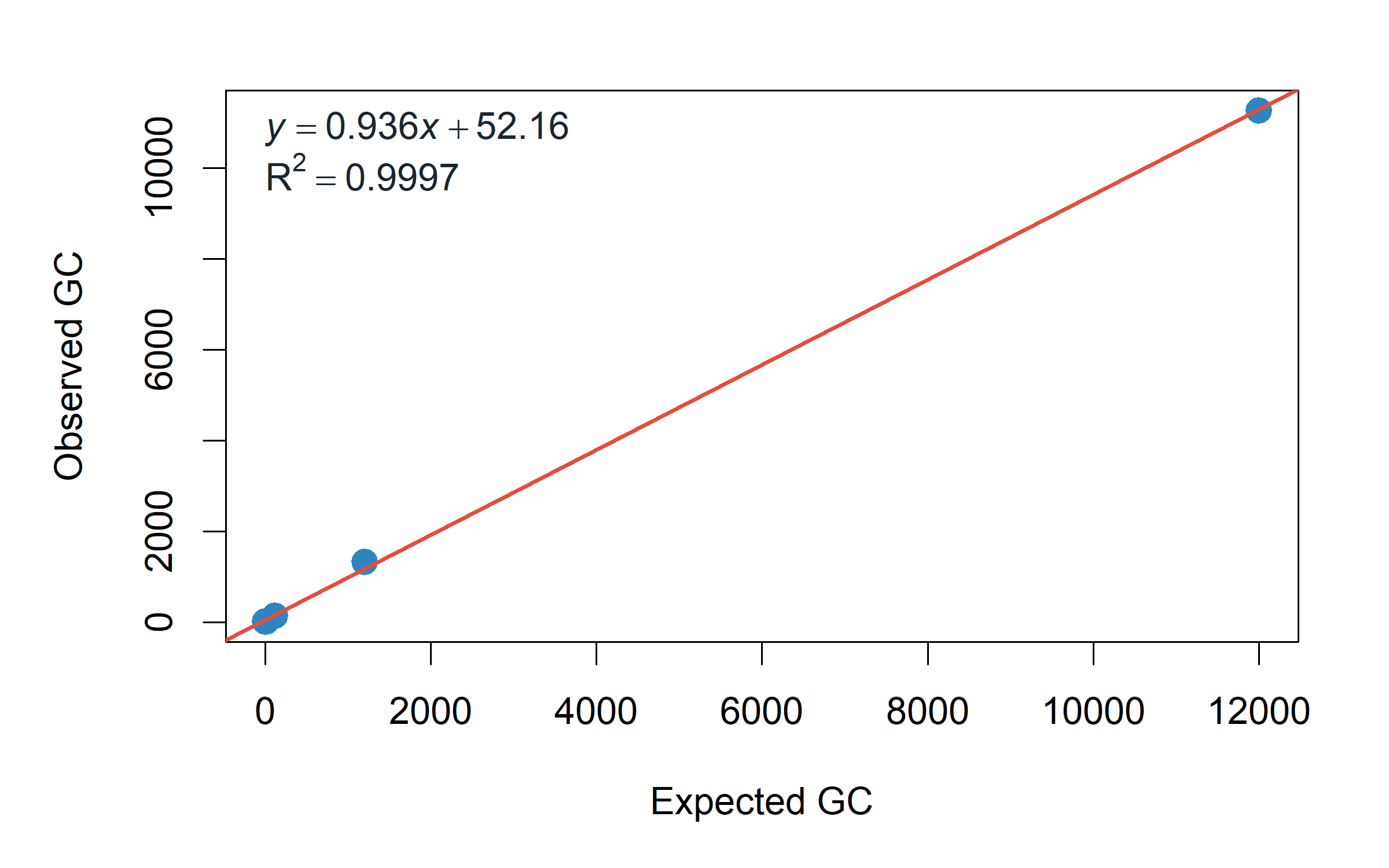


**Supplementary Figure 4:** Correlation between expected and observed SARS-CoV-2 genome copies measured by dPCR, showing high assay linearity (R² = 0.9997).


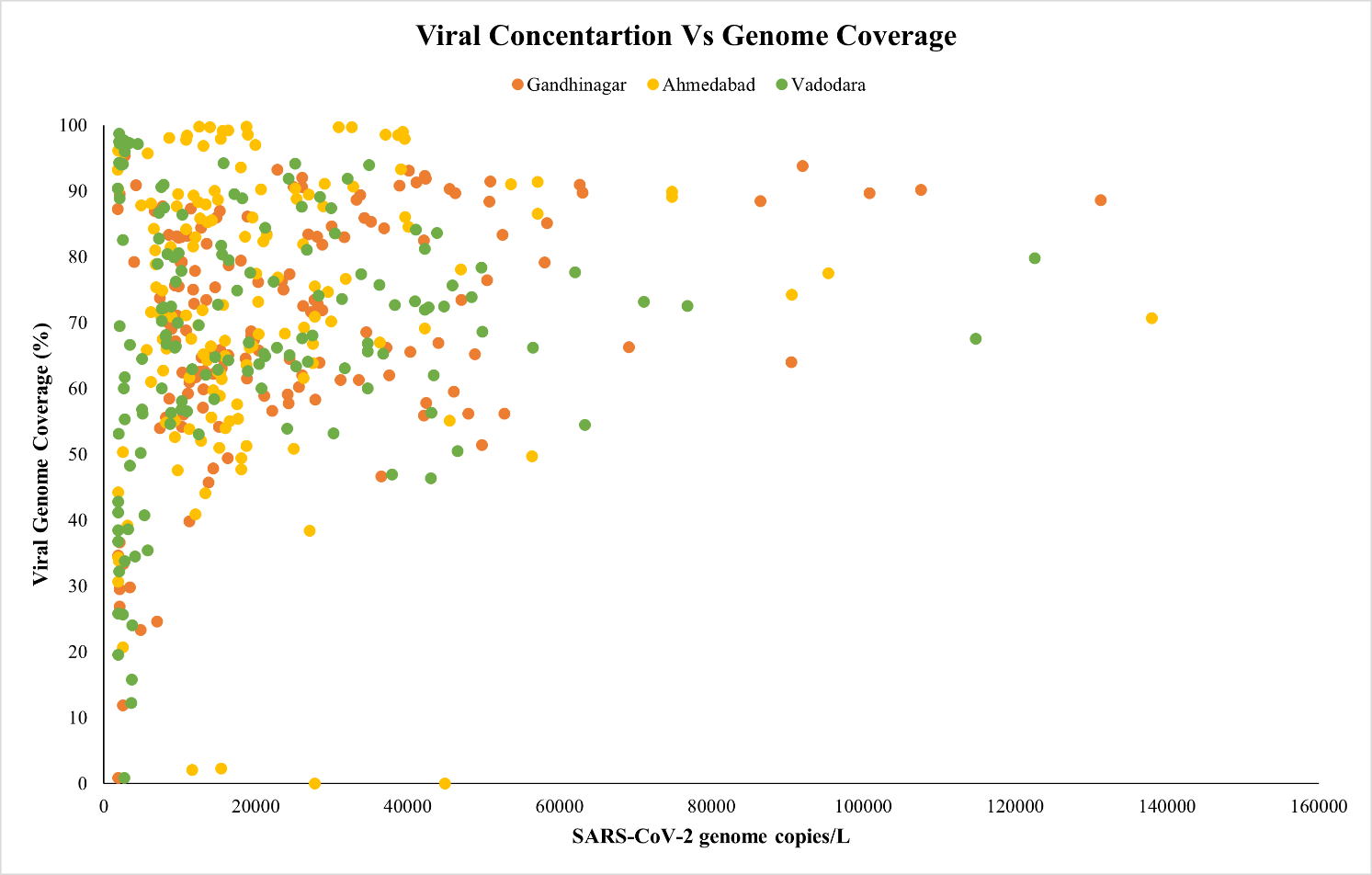


**Supplementary Figure 5:** Relationship between SARS-CoV-2 viral concentration and genome coverage in wastewater samples from Ahmedabad, Gandhinagar, and Vadodara.

## Supplementary Tables

Table S1 details the various sampling sites of Ahmedabad, Gandhinagar and Vadodara included in the study along with their latitude and longitude coordinates and the capacity of the sites.

**Table S1:** Sampling Sites of all three cities of the study.

| **City** | **Location** | **SPS/STP** | **Coordinates** | **Capacity** | **Catchment area** |
| --- | --- | --- | --- | --- | --- |
| **Ahmedabad** | Maninagar | SPS | 22.99776588, 72.59432447 | - | Maninagar area |
|  | Vinzol | STP | 22.938936, 72.646113 | - | from Kathwada to Vinzol |
|  | Ambawadi | SPS | 23.08636439, 72.63964825 | 285 MLD | Sardarnagar, Kubernagar, Kotarpur |
|  | Jamalpur | SPS | 23.01171175, 72.58198422 | - | Central zone sewage from Dariyapur, Shahpur, Raipur, jamalpur etc. |
|  | Pirana | STP | 22.97540366, 72.54951483 | 100 MLD | North, East and South zones of the city |
|  | Vasna | SPS | 22.98788033, 72.53446809 | - | West Zone from Kali Gam to Vasna |
|  | Vasna MLD | SPS | 22.98362835, 72.52550415 | - | New west zone from Gota to Sarkhej |
| **Gandhinagar** | Raysan | SPS | 23.159019, 72.651007 | 6 MLD | GUDA TP, TP-19, Randesan TP-18, Dholakuwa TP-15 |
|  | Kudasan | SPS | 23.183090, 72.628963 | 18-20 MLD | Kudasan TP-4,6, sargasan TP-7,8,9 |
|  | Sargasan | SPS | 23.195327, 72.621745 | 50-55 MLD | Sec 1-30 Gandhinagar, GIDC, Indroda, Dholakuwa |
|  | Jaspur | STP | 23.161372, 72.538694 | 65 MLD | Adalaj PS and outskirts |
|  | Vavol | SPS | 23.221903, 72.616784 | 6 MLD | GUDA TP area, and TP-13 |
| **Vadodara** | New Atladra | STP | 22.281773, 73.168141 | 43 MLD | - |
|  | Old Atladra | STP | 22.281773, 73.168141 | 43 MLD | Ataladra, Manjalpur, Tandalja, Gorwa, Sun Pharma |
|  | Bhayli | STP | 22.274658, 73.122501 | 45 MLD | Bhayli, Tandalja |
|  | 60 Kapurai | STP | 22.269903, 73.260023 | 60 MLD | Kapurai MPS, Khatamba, 43 MLD Kapurai |
|  | 43 Kapurai | STP | 22.269903, 73.260023 | 43 MLD | - |
|  | 21 Chhani | STP | 22.355547, 73.178449 | 21 MLD | Chhani, Nijampura, TP-13 Chhani |
|  | 50 Chhani | STP | 22.354827, 73.179024 | 50 MLD | Shastri bridge, new sama, old sama |
|  | Rajiv Nagar | STP | 22.325644, 73.231497 | 78 MLD | Amritnagar, Sardar state, Khodiyar nagar, Ajwa Road, New VIP Road |
|  | Sayaji Baug | STP | 22.316793, 73.193618 | 16 MLD | Fateh Ganj, Nagarwada, Bal bhavan |
|  | Tarsali | STP | 22.260664, 73.218479 | 52 MLD | GIDC domestic, Saradnagar, Danteshwar, Vuda na makan |
|  | Vemali | STP | 22.323834, 73.207372 | 2 MLD | Vemali area |
|  | Gajrawadi | STP | 22.287701, 73.21727 | 66 MLD | Gajarawadi, Bakrawadi, Pani gate |

**Table S2:** Primer and probe sequences used in Digital PCR

| **Name** | **Sequence (5′- 3′)** | **Final concentration per reaction [ μM]** |
| --- | --- | --- |
| nCoV _ N1 - F | GAC CCC AAA ATC AGC GAA AT | 0.8 |
| nCoV _ N1 - R | TCT GGT TAC TGC CAG TTG AAT CTG | 0.8 |
| nCoV _ N1 - P | FAM -ACCCCG CAT TAC GTT TGG TGG ACC -BHQ1 | 0.25 |
| nCoV _ N2 - F | TTA CAA ACA TTG GCC GCA AA | 0.8 |
| nCoV _ N2 - R | GCG CGA CAT TCC GAA GAA | 0.8 |
| nCoV _ N2 - P | FAM -ACA ATT TGC /ZEN / CCC CAG CGC TTC AG -IABkFQ | 0.25 |

**Table S3:** Common lineages found in the clinical as well as all three cities wastewater data set

| **Lineages** | | |
| --- | --- | --- |
| BA.2.38 | GE.1 | XBB.1.16.2 |
| XBB.2.3 | XBB.1.5 | XBB.1.6 |
| XBB.1.16.1 | XBB.1.16 | BL.1 |
| XBB.2.3.3 | XBB.2.3.2 |  |

**Table S4:** Unique lineages found in Clinical and wastewater sample data set

| **Wastewater Unique Lineages** | | | | | |
| --- | --- | --- | --- | --- | --- |
| XBB.1.16.4 | BA.2.76 | BA.2.74 | XBB.1.16.5 | BA.2.73 | XBB.1.22.2 |
| XBB.2.3.11 | XBB.2.4 | BA.2.37 | XBB.1.22 | BA.2.58 | BA.2.17 |
| XAS | BA.2.56 | BA.2.65 | BA.2.12 | XBB.1.16.17 | BA.2.10 |
| JN.1.1 | BA.2.1 | BA.2.12.1 | GE.1.1 | BL.1.3 |  |

| **Clinical Unique Lineages** | | | | | |
| --- | --- | --- | --- | --- | --- |
| BA.2.10.1 | JN.1 | FU.1 | XBB.1 | EG.5.2 | XBB.1.16.6 |
| XBB.1.5.32 | BA.2.38.1 | BA.2.86 | GJ.1.2 | HK.2 | XBB.1.16.13 |
| XBB.1.16.11 | FY.3.1 | EG.5.1.1 | FL.1.5.1 | EG.5.1 | XBB.2.3.4 |
| XBB.1.5.45 |  |  |  |  |  |
